# Phytochemical Composition, Bioactivities, and Medicinal Value of *Salvia nemorosa* L.

**DOI:** 10.3390/molecules31193522

**Published:** 2026-10-02

**Authors:** Ralitsa Parova, Stanislava Ivanova, Diana Karcheva-Bahchevanska, Borislava Lechkova, Kalin Ivanov, Iva Slavova, Zoya Dzhakova

**Affiliations:** 1 Department of Pharmacognosy and Pharmaceutical Chemistry, Faculty of Pharmacy, Medical University of Plovdiv, 4002 Plovdiv, Bulgaria; 2 Research Institute, Medical University of Plovdiv, 4002 Plovdiv, Bulgaria; 3 PERIMED-2, BG16RFPR002-1.014-0007, Central District, Vasil Aprilov Blvd. 15A, 4002 Plovdiv, Bulgaria; 4 Department of Chemical Sciences, Faculty of Pharmacy, Medical University of Plovdiv, 4002 Plovdiv, Bulgaria

**Keywords:** essential oil, natural products, phytochemistry, rosmarinic acid, *Salvia nemorosa*, woodland sage

## Abstract

*Salvia nemorosa* L. (Lamiaceae) is a perennial herb native mainly to Europe and Western Asia and has a fragmented but longstanding record of ethnomedicinal use. Although widely distributed, it remains a relatively underexplored member of the *Salvia* genus. Recent investigations have revealed that *S. nemorosa* is a rich source of bioactive secondary metabolites, particularly phenolic acids, flavonoids, diterpenoids, triterpenoids, megastigmane glycosides, and volatile terpenoids. Among these, rosmarinic acid, sagerinic acid, and caffeoylquinic acids are predominant constituents contributing to its pronounced antioxidant and anti-inflammatory activities. Essential oils of *S. nemorosa* are characterized by high levels of sesquiterpenes, such as β-caryophyllene and caryophyllene oxide, which exhibit notable antimicrobial, cytotoxic, and neuroprotective properties. A critical comparison of published data indicates pronounced quantitative and qualitative variability related to geographical origin, plant organ, developmental stage, cultivation conditions, and extraction/analytical procedures. Comparative studies with other *Salvia* species suggest that *S. nemorosa* can show high antioxidant or enzyme-inhibitory activity under particular experimental conditions, but these findings are not consistent across all assays or populations. Evidence for the biological activity of *S. nemorosa* itself is predominantly derived from in vitro experiments, while in vivo and clinical evidence remains lacking or very limited. Earlier isolation studies also demonstrate that the phytochemistry of the species extends beyond common phenolic compounds and volatile constituents, including quinones, diterpenoids, triterpenoids, and megastigmane glycosides. This review critically synthesizes the available evidence, with emphasis on phytochemical variability, plant-organ and geographical differences, comparisons with other *Salvia* species, and the limitations that currently prevent firm conclusions regarding therapeutic applications.

## 1. Introduction

The genus *Salvia* (Lamiaceae) is one of the largest genera of flowering plants, comprising nearly 1000 species distributed worldwide, many of which are well known for their aromatic properties and ethnomedicinal importance [1,2]. Species within this genus are characterized by a diverse phytochemical profile, including phenolic acids, flavonoids, diterpenoids, triterpenoids, and volatile terpenoids. However, *S. officinalis* L. and several other widely used medicinal *Salvia* species have been more extensively investigated than *S. nemorosa* L. for their biological and pharmacological properties [2,3].

*Salvia nemorosa* L. (woodland sage) is a perennial herb native to Europe and Western Asia that is distributed across a broad geographical range. *Salvia nemorosa (S. nemorosa)* attains a height between 30 and 50 cm and is characterized by dark purple stems bearing elongated, erect inflorescences composed of flowers that vary in coloration from white and pale pink to mauve, blue, and purple (Figure 1). The plant grows well in moderately moist soils and adapts to both full sun and partial shade. The flowering period typically extends from June to October [4].

Ethnobotanical and ethnomedicinal records, although scattered, indicate traditional use of *S. nemorosa* in several regions [2,3]. In Russia, woodland sage has been used as a substitute for *S. officinalis* [3]. In traditional Bulgarian medicine, it has been reported for stomach ache, diarrhea, hemorrhages, and furuncles [5]. In Turkish folk medicine, leaves have been used externally for hemostatic purposes [6], while in northwestern Iran, the species has been recorded among plants used traditionally in the management of diabetes [7]. These records indicate that the ethnomedicinal relevance of *S. nemorosa* should not be underestimated, although the evidence is heterogeneous and does not establish clinical efficacy. Despite the ethnomedicinal importance of *S. nemorosa*, the species has been investigated far less extensively than other medicinally important *Salvia* taxa [2,3,4].

Phytochemical investigations of *S. nemorosa* have identified a diverse array of secondary metabolites, including phenolic acids, flavonoids, diterpenoids, triterpenoids, megastigmane glycosides, and volatile terpenoids [3,8,9,10,11,12,13,14,15,16]. Phenolic constituents such as rosmarinic acid, sagerinic acid, and caffeoylquinic acids have been frequently identified as major constituents in *S. nemorosa* extracts, while its essential oils (EOs) contain numerous monoterpenoid and sesquiterpenoid constituents, including β-caryophyllene, germacrene D, caryophyllene oxide, spathulenol, and other constituents whose relative abundance differs among populations and plant organs [3,10,13,17].

The phytochemical diversity of *S. nemorosa* is further supported by isolation studies. Earlier investigations reported the isolation and structural characterization of specialized metabolites, including the diterpenoid nemorosin and the triterpenoid salvinemorol [15], as well as the megastigmane glycosides salvionosides A–C and other norisoprenoid glycosides [16]. More recently, bioassay-guided isolation led to the purification and structural characterization of clerodermic acid, a clerodane diterpenoid, which showed antiproliferative activity against A549 cells [18]. Thus, the phytochemical literature on *S. nemorosa* extends beyond the detection of common phenolic compounds and essential oil (EO) constituents and includes the isolation and characterization of structurally diverse specialized metabolites.

Importantly, the relative abundance of these compounds may vary according to geographical origin, plant organ, population, and analytical method used, which can make direct comparison between published studies challenging.

Interest in the biological properties of *S. nemorosa* has increased, with studies reporting antioxidant, antimicrobial, enzyme-inhibitory, cytotoxic, and anti-inflammatory effects in vitro [3,12,18,19,20,21,22,23,24,25,26,27,28,29,30,31]. However, these activities are strongly dependent on the plant material, extraction procedure, chemical composition, and experimental model.

The present review therefore distinguishes evidence obtained from whole extracts or EOs from effects demonstrated for isolated constituents. The aim of this review is to provide a critical analysis of the available literature on the phytochemical composition and biological activities of *S. nemorosa*, with particular emphasis on differences among plant organs, geographical populations, extraction procedures, and chemical profiles, as well as comparisons with other *Salvia* species. Research gaps and priorities for future phytochemical and pharmacological investigations are also identified.

## 2. Discussion

The literature identified in the present review is heterogeneous in design and analytical approach. Most available evidence concerns chemical profiling and in vitro assays. Rather than treating the literature as a simple catalog of compounds, the following sections compare chemical patterns across plant organs, geographical origins, extraction procedures, and populations, and then relate these patterns to reported biological activities.

A total of 29 studies were identified that investigated the chemical composition of *S. nemorosa* extracts and EOs, as well as their in vitro biological activity. Eleven studies examined the chemical composition of *S. nemorosa* extracts, 11 studies examined the chemical composition of its EOs, 15 in vitro studies evaluated the biological activity of *S. nemorosa* extracts, and 3 in vitro studies focused on its EOs. It should be noted that some studies address more than one research area. Current evidence is primarily derived from in vitro experiments, whereas in vivo investigations remain limited.

### 2.1. Chemical Diversity of Salvia nemorosa L.

Phytochemical investigations of *S. nemorosa* have indicated that the plant contains several classes of secondary metabolites, including phenolic acids, flavonoids, diterpenoids, triterpenoids, megastigmane and norisoprenoid derivatives, and volatile terpenoids [13]. Phenolic acids, particularly rosmarinic acid, sagerinic acid, and caffeoylquinic acids, are among the major constituents reported in *S. nemorosa* extracts, whereas the EOs are generally characterized by the presence of sesquiterpenes and, depending on geographical origin and plant organ, monoterpenes [13].

#### 2.1.1. Phytochemical Composition of *S. nemorosa* Extracts

Phenolic acids constitute an important part of the chemical profile of *S. nemorosa*. Rosmarinic acid has been repeatedly reported as a major component, along with sagerinic acid, caffeoylquinic acids, caffeic acid, chlorogenic acid, ferulic acid, and *p*-coumaric acid. However, the relative abundance of these compounds in extracts can vary considerably depending on geographical origin, plant organ, developmental stage, extraction procedure, and analytical methodology.

Rosmarinic acid (Figure 2) is an ester formed between caffeic acid and 3,4-dihydroxyphenyllactic acid. The compound is associated with a wide range of biological activities, including antiviral, antibacterial, anti-inflammatory, and antioxidant effects [32]. Rosmarinic acid represents one of the predominant phenolic constituents identified in *S. nemorosa*, highlighting the plant’s potential as a promising natural source of this compound. Moreover, Khoshsokhan et al. developed an efficient protocol for the production of rosmarinic acid in *S. nemorosa* [33]. In another study, the combination of gamma irradiation and multi-walled carbon nanotubes elicitation was shown to enhance the biosynthesis of phenolic acids in *S. nemorosa* cell cultures, particularly rosmarinic acid, salvianolic acid B, cinnamic acid, and ferulic acid, suggesting a promising strategy for increasing the yield of valuable secondary metabolites [34].

More recent evidence further demonstrates that the phenolic profile of *S. nemorosa* can be modified by biotic interactions and elicitation treatments. Fotovvat et al. investigated the combined effects of arbuscular mycorrhizal fungi (AMF) and chitosan on *S. nemorosa* and reported changes in total phenolic and flavonoid contents, phenylalanine ammonia-lyase activity, and the accumulation of rosmarinic acid and quercetin in leaves and roots. The responses depended on the AMF species, chitosan concentration, and plant tissue, further supporting the influence of cultivation and elicitation conditions on phenolic accumulation in *S. nemorosa* [35].

Caffeoylquinic acids (CQAs), ester derivatives of caffeic acid and quinic acid, exhibit antioxidant activity through hydrogen atom and electron donation. Computational studies indicate that CQAs may effectively scavenge hydroperoxyl radicals and inhibit xanthine oxidase through stable enzyme–ligand complexes. The dual mechanism of action suggests the potential of CQAs as natural antioxidants and may contribute to the pharmacological relevance of *Salvia* species [36]. These acids are also recognized for their anti-inflammatory, neuroprotective, antidiabetic, and anticancer effects [37,38,39].

Sagerinic acid is another phenolic compound identified in various *Salvia* species, including *S*. *nemorosa* L. It is a dimeric derivative of rosmarinic acid formed through a photochemical cycloaddition reaction [40]. The acid has demonstrated significant antioxidant properties and has inhibitory effects on enzymes like acetylcholinesterase, indicating possible neuroprotective and anti-inflammatory effects in herbal extracts [41]. Studies on the phytochemical diversity of *S. nemorosa* extracts are significantly limited and are presented in Table 1.

As summarized in Table 1, the composition of *S. nemorosa* extracts differs according to geographical origin, plant organ, extraction procedure, and analytical methodology.

Substantial quantitative variation may occur even among populations collected within a relatively restricted geographical area. Moshari-Nasirkandi et al. investigated 70 *S. nemorosa* populations from 54 habitats in West Azerbaijan Province, Iran. The total phenolic content ranged from 33.87 mg GAE/g to 66.24 mg GAE/g, while the total flavonoid content ranged from approximately 9.0 mg QE/g to 13.8 mg QE/g dry weight [13]. UHPLC-HRMS analysis identified rosmarinic acid, sagerinic acid, and caffeoylquinic acids among the major phenolic constituents [13].

Rosmarinic acid was also reported as the predominant phenolic constituent in several studies using different geographical materials and extraction approaches. Bahadori et al. reported a concentration of 7584 µg/g extract for *S. nemorosa*, with the methanolic extract showing the highest total phenolic and flavonoid contents among the solvents investigated [3]. In samples from the Romanian flora, Coisin et al. reported 268.79 mg/100 g dry plant material of rosmarinic acid, whereas caffeic acid and chlorogenic acid occurred at considerably lower concentrations of 4.76 mg/100 g and 4.75 mg/100 g, respectively. Apigenin (25.92 mg/100 g) and luteolin (9.86 mg/100 g) were also detected [14]. Similarly, Bălăşoiu et al. identified rosmarinic acid as the major phenolic acid in aerial-part extracts, with a concentration of 1692.434 µg/g dry weight, followed by chlorogenic acid at 56.316 µg/g dry weight [19].

Although these studies identify rosmarinic acid as an important constituent, the reported concentrations could not be compared as absolute values, as the investigations differed in plant material, extraction procedures, analytical methods, and units of expression. Moreover, rosmarinic acid was not a predominant component in all samples. For example, in aerial-part extracts of *S. nemorosa* from Moldova, ferulic acid and *p*-coumaric acid were reported as the main quantified phenolic acids, at 182.1 µg/g and 146.4 µg/g dry weight, respectively. Rutin, luteolin, and apigenin were the principal flavonoid constituents detected under the conditions investigated [23].

The influence of the extraction procedure is of particular importance, and this is evident in the study by Ivanov et al., in which fourteen extracts of *S. nemorosa* inflorescences were prepared using different solvents, including ethanol–water, ethanol, dichloromethane–hexane, and butanol. The different extraction procedures resulted in extracts with distinct biological activities, suggesting that the polarity of the solvent may affect both the chemical composition of the extracts and their biological activity [12].

Differences in the accumulation of phenolic compounds in plant organs have also been reported. Methanol extracts of cultivated *S. nemorosa,* prepared separately from leaves, stems, flowers, and roots, showed total phenolic contents ranging from 7.3 mg GAE/g to 39.8 mg GAE/g extract, with the highest value recorded for flowers. The total flavonoid contents ranged from 68.8 mg QE/g to 141.9 mg QE/g extract, and leaves showed the highest value [19]. In another study, the total polyphenol levels were higher in leaves than flowers, with values of 11.96 mg GAE/g and 8.55 mg GAE/g fresh weight, respectively [20]. Besides plant organs, differences related to developmental stage and growing conditions may also contribute to the observed variability between samples and therefore should be taken into account when interpreting phytochemical data.

The phytochemical profile of *S. nemorosa* is relatively consistent qualitatively but shows greater quantitative variation. In addition to the compounds discussed, the species also contains characteristic metabolites. Ulubelen et al. reported the isolation of the new diterpenoid nemorosin and the new triterpenoid salvinemorol from the aerial parts of *S. nemorosa*. Both compounds were characterized structurally using spectroscopic methods [15]. Subsequently, Takeda et al. announced three new megastigmane glycosides, salvionosides A–C, together with several known norisoprenoid glycosides from the aerial parts of the species [16]. More recently, bioassay-guided fractionation of the aerial parts of *S. nemorosa* resulted in the isolation and structural characterization of clerodermic acid, a clerodane diterpenoid with demonstrated antiproliferative activity against A549 cells [18]. This finding further supports the diterpenoid diversity of *S. nemorosa* and highlights the potential biological relevance.

Overall, *S. nemorosa* contains a diverse range of phenolic and other secondary metabolites, with rosmarinic acid being one of the most consistently reported compounds. However, differences between plant materials and the limited number of isolation studies suggest that the chemical composition of the species is still not fully understood.

#### 2.1.2. Phytochemical Composition of *S. nemorosa* Essential Oils

The essential oil (EO) of *S. nemorosa* represents a complex mixture of volatile terpenoid compounds and fatty acids. Among the major constituents, sesquiterpenes predominate, including β-caryophyllene, caryophyllene oxide, germacrene D, and spathulenol, along with monoterpenes such as α-thujene and γ-terpinene. Hexadecanoic acid (palmitic acid) is also present as a non-volatile fatty acid component (Table 2).

The data summarized in Table 2 demonstrate pronounced qualitative and quantitative variability in *S. nemorosa* EO composition. Although several constituents, including β-caryophyllene, caryophyllene oxide, germacrene D, spathulenol, and sabinene, are repeatedly reported in the analyzed samples, their relative content can vary substantially. These differences could be related to geographical origin, climatic conditions, plant organ, and agronomic practices.

Environmental conditions can also modify the physiological and chemical characteristics of *S. nemorosa*. Sharifi and Shirani Bidabadi reported that salinity induced oxidative stress in *S. nemorosa*, whereas exogenous strigolactone enhanced antioxidant defense, alleviated the negative effects of salinity on gas exchange, and increased EO yield. These findings indicate that environmental stress can influence not only the physiological status of the species but also the production of its volatile fraction [47].

A similar influence of environmental conditions was observed under water deficit. Bidabadi et al. investigated *S. nemorosa* under different irrigation regimes and melatonin treatments and identified β-caryophyllene, germacrene-B, spathulenol, and *cis*-β-farnesene as the principal EO constituents. Both irrigation regime and melatonin treatment affected EO yield and the relative abundance of the major constituents, with mild water stress and melatonin treatment producing distinct changes in the volatile profile. These results provide further evidence that the EO composition of *S. nemorosa* is environmentally responsive rather than fixed [48].

Taken together, these findings indicate that the EO composition of *S. nemorosa* is influenced by both environmental conditions and the characteristics of the plant material. Therefore, the available evidence does not support a single EO profile that can be considered representative of the species. This highlights the need for appropriate standardization when studying and comparing *S. nemorosa* EOs.

Methodological differences should also be taken into account when comparing these studies. Most of them used GC-MS, while some also used GC-FID, and the analyses involved different plant organs and plant materials. Such methodological and sampling differences may influence the EO composition and complicate direct comparison of percentage results.

The available data further demonstrate that variation occurs not only between geographical regions but also among populations and plant organs. Studies from Austria and Iran demonstrate the influence of plant organ and geographical origin, whereas Bulgarian data indicate that notable differences may also arise between populations within the same country. Across Bulgarian populations, germacrene D predominated in three populations (25.74–41.34%), whereas sabinene was the major constituent in the Ivan Vazovo population (21.89%) [43]. Austrian material demonstrated clear organ-dependent differences: β-caryophyllene and germacrene D predominated in leaf oils, sabinene in flower oils, and hexadecanoic acid in several stem oils [10]. Serbian populations likewise differed in the relative abundance of caryophyllene oxide, spathulenol, β-caryophyllene, and germacrene D [8,9]. Iranian populations showed still greater variation, including spathulenol-, thymol-, (*E*)-caryophyllene-, and caryophyllene oxide-rich profiles [17]. These observations support pronounced infraspecific chemical variability and raise the possibility of chemotypes, although individual profiles should not be designated as confirmed chemotypes without standardized sampling and multivariate analysis.

A recent comparative study of five Iranian *Salvia* species cultivated under uniform ecological conditions further supports the variability of the volatile profile of *S. nemorosa*. Mohammadi et al. identified 23 constituents in *S. nemorosa* EO, with spathulenol (23.21%), *trans*-caryophyllene (21.92%), caryophyllene oxide (17.18%), and bicyclogermacrene (7.75%) as the major components. The species were cultivated under comparable environmental conditions, suggesting that differences in EO composition among *Salvia* species may also reflect species-specific characteristics rather than being attributed solely to contrasting environmental conditions [49].

Importantly, the biological significance of individual EO constituents should not be equated with the biological activity of the complete *S. nemorosa* EO. Most pharmacological information on β-caryophyllene, caryophyllene oxide, germacrene D, spathulenol, sabinene, and thymol derives from studies of isolated compounds or other plant species. Accordingly, these compounds are best considered plausible contributors to, rather than direct proof of, the activities reported for *S. nemorosa* EOs.

#### 2.1.3. Comparative Context Within the Genus *Salvia*

Comparative studies provide an important context for interpreting the chemical and biological profile of *S. nemorosa*. In a study of 32 *Salvia* species from Iran, *S. nemorosa* from West Azerbaijan showed high total phenolic and flavonoid contents (119.3 mg GAE/g and 51.8 mg CE/g extract, respectively), although it was not the richest species overall [22]. Studies comparing several *Salvia* species also reported high antioxidant activity for *S. nemorosa* under specific assay conditions, including higher activity than *S. officinalis* and *S. sclarea* in selected FRAP or radical-scavenging assays [20,25,26,27,28]. Conversely, comparative studies demonstrate that antioxidant and enzyme-inhibitory activities vary widely among *Salvia* species and among populations of the same species [21,22,26,27,30]. For α-glucosidase inhibition, for example, *S. nemorosa* samples collected from four Iranian regions ranged from 23.5% to 70.1% inhibition at 1000 µg/mL [30]. Thus, the available evidence supports biological potential under defined experimental conditions but does not establish a unique pharmacological superiority of *S. nemorosa* over other medicinal *Salvia* species.

### 2.2. Biological Activity of Salvia nemorosa L.

The biological activity of *S. nemorosa* has been studied, mainly using its extracts and essential oils. The reported effects include antioxidant, antimicrobial, enzyme-inhibitory, cytotoxic, and wound-healing activities. However, the observed effects differ depending on the plant material used, extraction method, concentration, and experimental conditions.

#### 2.2.1. Biological Activity of *Salvia nemorosa* Extracts

*S. nemorosa* extracts have received particular attention due to their high content of phenolic compounds, especially rosmarinic acid, sagerinic acid, and caffeoylquinic acids. These compounds may contribute to some of the biological effects reported for the extracts, especially their antioxidant, antimicrobial, and anti-inflammatory properties (Table 3).

##### Antioxidant Activity

A number of methods have been used to assess the antioxidant potential of *S. nemorosa*, including 2,2-diphenyl-1-picrylhydrazyl radical (DPPH·) scavenging capacity, 2,2′-azinobis (3-ethylbenzothiazoline-6-sulphonic acid) (ABTS) assay, NO scavenging activity, and Ferric Ion Reducing Antioxidant Power Assay (FRAP). *S. nemorosa* demonstrates remarkable antioxidant properties due to the presence of phenolic compounds and diterpenoids, which play an important role in protecting against free radicals [50].

By studying the antioxidant properties of methanol extracts of eight species of *Salvia* cultivated under farm conditions, Esmaelli et al. revealed that the stem of *S. nemorosa* exhibited one of the highest antiradical scavenging activities (IC_50_ = 14.3 μg/mL) when tested against DPPH radical, along with those of roots of *S. macrosiphon* and *S. sclarea* (IC_50_ = 10.9 and 14.9 μg/mL, respectively), and leaves of *S. atropatana* (IC_50_ = 14.0 μg/mL) [42]. These results are in accordance with those obtained in the study of Bălăşoiu et al., where 70% methanol extract of *S. nemorosa* collected from the Oltenia region flora (Romania) demonstrated the strongest radical scavenging ability (15.84 μg/mL) compared to *S. aethiopis* L., *S. glutinosa* L., *S. sclarea* L., and *S. verticillata* L. [19]. In contrast, Firuzi et al. reported that a significantly higher concentration of the *S. nemorosa* extract obtained by methanol maceration was required to achieve 50% scavenging of DPPH radicals (IC_50_ 138.43 μg/mL) [28].

In another comparative study, it was found that among 15 species of the Lamiaceae family, cultivated in the climatic conditions of Iran, *S. nemorosa* showed the highest antioxidant potential determined by the FRAP method. Furthermore, the leaf extract of *S. nemorosa* exhibited a higher antioxidant capacity (2.92 mmol Fe/100 g fresh weight) than the flower extract (2.66 mmol Fe/100 g fresh weight) [20].

The observed difference in the antioxidant properties of *S. nemorosa* extract may be due to the different content of phenolic compounds. Kamatou et al. point out that the good antioxidant activity of *Salvia* species is related to the presence of rosmarinic acid, caffeic acid, carnosol, and derivatives (carnosic acid) [51]. Moreover, there is evidence in the literature of a positive correlation between antioxidant test results and the amount of total phenols [13]. For example, Mervic et al. showed that ferulic acid, caffeic acid, and rosmarinic acid correlate strongly with DPPH scavenging activity (r = 0.88, 0.86, and 0.54, respectively) [21].

It is well established that the phenolic profile of plants is influenced by geographical origin, climatic conditions, parts of plants, solvent, and methods used for extraction [52]. In this regard, a 2017 publication showed that the polarity of the solvent influences the concentration of extracted phenolic compounds from *S. nemorosa*, and hence its antioxidant potential [3]. In this study, the aerial parts of *S. nemorosa* were extracted with *n*-hexane, dichloromethane, and methanol, and it was found that the methanol extract demonstrated strong radical scavenging properties (IC_50_ 82.1 μg/mL), while the other extracts showed unsatisfactory activity (>500 μg/mL). Also, the IC_50_ values for the EOs obtained from leaves and flowers of *S. nemorosa* were higher than 500 μg/mL. The observed trend is also confirmed when determining the reducing power of the extracts using the FRAP assay. The methanol extract showed the strongest reducing activity (SRP 0.99), followed by the leaf and flower EO (SRP 0.77 and 0.67, respectively), which the authors attributed to the higher content of phenols and flavonoids in the extract and spathulenol in the EOs [3].

##### Antidiabetic Activity

Several in vitro studies have shown that *Salvia* species lower blood sugar [53,54]. In this regard, Mervic et al. evaluated the α-glucosidase inhibitory activity of seven wild-growing *Salvia* species (*S. fruticosa*, *S. glutinosa*, *S. nemorosa*, *S. officinalis*, *S. pratensis*, *S. sclarea*, and *S. verticillata*) from the Mediterranean area [21]. The results of the study showed that all sage extracts at the tested concentrations (400–6400 µg/mL) were capable of blocking the enzyme. Specifically, *S. fruticosa* and *S. officinalis* exhibited the strongest inhibitory activity with IC_50_ values of 5291.51 and 4451.85 µg/mL, respectively, which were not statistically different from those achieved by rosmarinic acid. At the highest concentration tested, the ethanol extract of *S. nemorosa* leaves inhibited α-glucosidase by 39.3%, which was more than 2 times lower than the antidiabetic drug acarbose (89.1%) [21].

In another study, Bahadori et al. reported that the methanol extract of the aerial parts of *S. nemorosa* showed strong inhibitory activity on α-glucosidase (IC_50_ 19 μg/mL), which was comparable to that of acarbose (16 μg/mL) [3].

Also, 18 methanolic extracts of 13 Lamiaceae plants (roots and aerial parts) demonstrated α-amylase inhibitory activity ranging from 0.135 to 0.291 mmol acarbose equivalents/g (ACAEs/g) extract and α-glucosidase inhibitory activity ranging from 1.25 to 6.46 mmol ACAEs/g extract [29]. Among the extracts tested, only *Scutellaria tomentosa* roots showed a better ability (0.291 mmol ACAEs/g) to slow down the breakdown of starch to glucose. A lower inhibitory potential was observed for the *S. nemorosa* leaf extract, with an activity of 0.170 mmol ACAEs/g extract. In the α-glucosidase assay, both the roots and leaves of *S. nemorosa* showed moderate inhibitory activity with values of 2.72 and 2.96 mmol ACAEs/g extract [29].

Furthermore, the impact of extraction solvents on the inhibitory potential of *S. nemorosa* inflorences against α-amylase and α-glucosidase has been reported [12]. The results revealed that sage extracts, obtained using 100% butanol and a 70:30 mixture of ethanol and butanol, exhibited the strongest α-amylase inhibitory effect with IC_50_ values of 0.44 mg/mL and 0.66 mg/mL, respectively. It was observed that the dichloromethane extracts were the least active against α-amylase. On the other hand, the ethanol-water mixture (50:50) proved to be the most effective (IC_50_ 0.18 mg/mL) for extracting α-glucosidase inhibitors from *S. nemorosa* [12].

A comparative study on 14 Turkish *Salvia* species revealed that there is a strong correlation between the content of the triterpenoids ursolic acid and oleanolic acid and the inhibitory activity of sage extracts against α-glucosidase [55]. A 2019 study of extracts from 14 different plants of the *Salvia* genus found that the rosmarinic acid content determined in all samples was related to the ability of sage extracts to inhibit the action of the enzyme α-glucosidase. Furthermore, the best inhibitory activity was obtained from *S. caespitosa* (IC_50_ 5.51 µg/mL), which is characterized by the highest content of rosmarinic acid (18.7 mg/g DW) and is the only extract containing carnosic acid (11 mg/g DW) [56]. This is in line with the results achieved by Shojaeifard et al., who reported that the main constituents in sage extracts responsible for their antidiabetic activity were rosmarinic acid, carnosol, and carnosic acid [30]. In this study, hydromethanol extracts of 50 *Salvia* samples belonging to 32 different species were examined for their α-glucosidase properties. Among the extracts studied, *S. santolinifolia* was the most effective inhibitor of α-glucosidase, followed by *S. multicaulis* and *S. eremophila* with IC_50_ values of 26.2 µg/mL, 30.7 µg/mL, and 33.2 µg/mL, respectively. The methanol extracts of *S. nemorosa* collected from four different regions in Iran exhibited α-glucosidase inhibitory activity ranging from 23.5% to 70.1%. Among all the *S. nemorosa* samples, the one from the West Azerbaijan region showed the highest antidiabetic effect (70.1% inhibition at a concentration of 1000 µg/mL), which was closest to that of the reference standard, acarbose (87.8%). In addition, the presence of salvianolic acids and abietane diterpenoids may play a key role in the inhibitory activity of the *Salvia* species [30].

Other antihyperglycemic compounds from *Salvia* species can be flavonoids: apigenin, luteolin, and quercetin [29]. Therefore, it can be assumed that the strong inhibitory potential of plants of the genus *Salvia* may be due to the presence of rosmarinic acid together with terpenoids.

##### Antimicrobial Activity

Plant-derived preparations have attracted considerable attention in the context of increasing antimicrobial resistance. Recent studies have highlighted the antibacterial potential of plant extracts and EOs, including their activity against resistant and clinically relevant microorganisms [57,58,59]. *Salvia* species produce a wide range of phytoconstituents that may contribute to antibacterial and antifungal effects [60,61,62].

The antimicrobial potential of *S. nemorosa* has been investigated using different plant materials, extraction procedures, different strains of bacteria, and bioassays, with substantial variation in the reported antimicrobial activity depending on these factors. Overall, the available studies suggest that Gram-positive bacteria tend to be more susceptible to *S. nemorosa* extracts than Gram-negative bacteria.

In an investigation of several *Salvia* species from Iran, the antimicrobial effect of *S. nemorosa* was comparatively limited, with activity reported only against *S. typhi* and a MIC of 10 mg/mL [28]. In contrast, Bahadori et al. reported pronounced activity of *S. nemorosa* EOs and extracts, particularly against Gram-positive bacteria. The EO obtained from the leaves produced a 33.7 mm inhibition zone against *Staph. aureus*, while the methanolic extract and flower EO produced inhibition zones of 33.0 and 32.0 mm, respectively. The methanolic extract and flower EO also showed inhibitory activity against *C. albicans* [3].

Evidence from Eastern European *Salvia* species further supports the antimicrobial potential of *S. nemorosa*, particularly against fungal pathogens. Using 80% ethanol extracts of the aerial parts, Luca et al. found that *S. nemorosa* and *S. pratensis* were among the most active species against *C. albicans*, with a MIC of 1.25 mg/mL. In contrast, the same *S. nemorosa* extract showed lower activity against the bacterial strains tested, including *Staph. aureus* and *S. pneumoniae* [25].

More detailed evidence for *S. nemorosa* was provided by Ivanov et al., who demonstrated that the extraction solvent significantly affected the antibacterial activity of inflorescence extracts. Butanol and butanol/ethanol extracts displayed the strongest effects against the tested bacteria, including *Staph. lugdunensis*, *Staph. epidermidis*, and *Proteus vulgaris*. *P. vulgaris* was particularly susceptible, with an MIC value of 0.031 mg/mL for selected extracts. Activity against *Staph. lugdunensis* was also evident, whereas *Staph. epidermidis* was comparatively resistant. The same study further showed that several *S. nemorosa* extracts substantially reduced *Staph. lugdunensis* biofilms. The antifungal activity also varied depending on the extraction procedure. *C. parapsilosis* was particularly susceptible to the butanol and butanol/ethanol extracts, with MIC values of 0.25 mg/mL, whereas *C. albicans* showed substantially greater resistance, with MIC values reaching 4 to >8 mg/mL. Ethanol/water extracts were particularly active against *C. tropicalis*, exhibiting a MIC of 0.5 mg/mL. The observed antifungal activity showed associations with the content of phenolic acids, glycosides, and several flavonoid aglycones, including luteolin, apigenin, and kaempferol. However, the lack of a consistent relationship between individual compounds and antibacterial activity suggests that multiple constituents may contribute to the antimicrobial effects of *S. nemorosa* extracts [12].

The influence of preparation type on antimicrobial activity is further illustrated by hydrolate-based preparations. Ürgeová et al. evaluated microwave-assisted hydrolates prepared from fresh leaves of several *Salvia* species and found that *S. nemorosa* hydrolate exhibited partial inhibitory activity against the tested bacterial strains, whereas the antibacterial effect differed among the investigated *Salvia* species. This finding further indicates that antimicrobial responses of *S. nemorosa* should be interpreted in relation to the preparation method and chemical composition rather than attributed to the species independently of the tested preparation [63].

In a comparative study using aqueous, methanolic, and hydroalcoholic preparations, the aqueous *S. nemorosa* extract produced a pronounced inhibitory effect against *P. aeruginosa*, with an inhibitory zone of 26 ± 1 mm. The study suggested that the antimicrobial response was influenced not only by the extraction solvent but also by the amount of extract applied. The antimicrobial activity of *S. nemorosa* may be partly related to its relatively high saponin content, but it is likely to reflect the combined contribution of other secondary metabolites as well [24].

Overall, the available evidence indicates that *S. nemorosa* exhibits broad antimicrobial activity, particularly against Gram-positive bacteria and several *Candida* species, although its efficacy varies considerably depending on the extract preparation. Differences among reported MIC values likely reflect variations in plant material, geographical origin, extraction solvent, chemical composition, microbial strain, and methodology. Nevertheless, the activity observed in selected extracts supports further investigation of *S. nemorosa* as a source of antimicrobial compounds.

##### Cytotoxic and Antiproliferative Activity

The cytotoxic and antiproliferative properties of *S. nemorosa* have received considerably less attention than its antioxidant and antidiabetic activities. An indication of cytotoxic potential was obtained by Bahadori et al. using the Brine shrimp lethality assay. Extracts (*n*-hexane, DCM, and methanol) from the aerial parts of *S. nemorosa* collected in Iran, as well as EOs from flowers and leaves, were evaluated in the assay. The *n*-hexane extract showed the highest toxicity, with an LC_50_ value of 194.8 ± 14.6 µg/mL, whereas the other extracts demonstrated moderate effects, and the EOs showed no substantial lethality [3].

Firuzi et al. investigated methanol, 80% methanol, and DCM extracts of *S. nemorosa* from Iran using the MTT assay and evaluated the effects against HL-60, K562, and MCF-7 cells. Among the tested preparations, only the 80% methanol extract showed a cytotoxic effect against K562 cells (IC_50_ 87.0 ± 6.7 µg/mL) [28].

In a more detailed investigation of the antiproliferative potential of the species, *S. nemorosa* extracts were subjected to activity-guided fractionation, leading to the isolation of the clerodane diterpenoid clerodermic acid. Its antiproliferative activity was evaluated against A549 cells using an MTT assay. Clerodermic acid exhibited a pronounced effect, with an IC_50_ value of 35 μg/mL. Its activity was further supported by DNA fragmentation analysis, DAPI staining, cell cycle analysis, and annexin V/PI detection of apoptosis. The results provided evidence that the reduction in cell viability of A549 cells was accompanied by genotoxic effects, chromatin condensation, and apoptotic cell death. Moreover, treatment of hypoxic A549 cells with clerodermic acid reduced HIF-1α expression, as demonstrated by quantitative qPCR analysis. These findings identify clerodermic acid as a promising antiproliferative compound from *S. nemorosa*, warranting further investigation in vivo [18].

The cytotoxicity of *S. nemorosa* extracts has also been examined in a human keratinocyte model. Ivanov et al. prepared several extracts from *S. nemorosa* inflorescences collected in Serbia using different solvents and evaluated their effects on HaCaT cells. Only four of the extracts showed cytotoxic effects, with IC_50_ values in the range of 169.01–299.15 μg/mL, whereas the other extracts showed no substantial effect. A positive correlation was suggested between the cytotoxic effect and the content of rutin, rosmarinic acid, and phenolic acids in the extracts [12].

In contrast to the cytotoxic effects described above, Luca et al. reported a comparatively limited effect of an ethanolic extract from aerial parts of *S. nemorosa* collected in Moldova against MCF-7 and MDA-MB-231 cells. The treatments with the extracts did not result in a significant reduction in cell viability. The authors emphasized that the extract polarity, chemical composition, applied concentration, and treatment duration may affect the cytotoxic activity of the extracts [25].

##### Wound-Healing and Other Biological Effects

The wound-healing potential of *S. nemorosa* remains relatively underexplored. Ivanov et al. investigated the wound-healing properties of 14 extracts of *S. nemorosa* inflorescences using the scratch-wound healing assay. The most pronounced effect was observed for the ethanol:butanol 50:50 extract, achieving 43.17% wound closure. The authors suggested that the activity may result from synergistic interactions among the extract compounds rather than the action of a single constituent [12].

The available in vitro evidence indicates that *S. nemorosa* extracts can exhibit antioxidant, antimicrobial, enzyme-inhibitory, wound-healing-related, and, in selected cases, cytotoxic activities. However, the magnitude and direction of the effects vary with extraction solvent, plant organ, geographical origin, and assay. For example, methanolic extracts have shown strong DPPH activity in some studies, whereas other comparative investigations placed *S. nemorosa* below other *Salvia* species [3,19,25,26,27,28]. Antimicrobial effects also range from measurable inhibition of selected Gram-positive bacteria and *Candida* species to weak or absent activity against other organisms [24,25,28]. Bioassay-guided isolation of clerodermic acid from *S. nemorosa* is an important species-specific example: the isolated diterpenoid showed antiproliferative activity against A549 cells, but this finding should not be generalized to the whole plant extract [18]. Overall, the heterogeneity of experimental systems prevents direct ranking of the extracts.

#### 2.2.2. Biological Activity of *S. nemorosa* Essential Oils

*S. nemorosa* EOs have also shown antioxidant and antimicrobial activity in vitro (Table 4). The reported effects differ between samples and should be interpreted alongside the corresponding EO composition. In particular, the marked variation in β-caryophyllene, caryophyllene oxide, germacrene D, spathulenol, sabinene, and other constituents provides a plausible explanation for some inter-study differences, but direct composition-activity relationships remain insufficiently established.

Taken together, the EO studies demonstrate measurable in vitro antioxidant and antimicrobial effects, but the evidence does not support a uniform pharmacological profile. Future work should report complete EO composition together with standardized bioassay conditions so that chemical variability can be quantitatively related to biological endpoints.

The variability in EO composition depending on geographic origin and extraction method can significantly influence biological performance, emphasizing the importance of chemical standardization in future investigations. Overall, *S. nemorosa* demonstrates multifunctional bioactivity, with synergistic effects between its metabolites suggesting valuable therapeutic potential for oxidative stress, infection, and inflammation-related disorders.

### 2.3. Future Perspectives

The phytochemical literature demonstrates that *S. nemorosa* has been investigated beyond routine phenolic and EO profiling. Early isolation studies reported a new quinone from the species [65], while subsequent work identified the new diterpenoid nemorosin and triterpenoid salvinemorol from aerial parts [15]. Takeda et al. isolated the new megastigmane glycosides salvionosides A–C together with known norisoprenoid glycosides [16], and bioassay-guided fractionation later yielded clerodermic acid, a clerodane diterpenoid with antiproliferative activity against A549 cells [18]. These results demonstrate that *S. nemorosa* is a source of structurally diverse specialized metabolites, while the relatively limited number of studies focused on their isolation suggests that the species’ metabolic diversity has not yet been fully elucidated. Therefore, further investigations of underexplored plant organs, populations, developmental stages, and environmental conditions could reveal additional compounds and clarify the factors underlying chemical variation within the species.

The biological evidence for *S. nemorosa* remains predominantly based on in vitro experiments. To date, evidence directly demonstrating the pharmacological efficacy of *S. nemorosa* in vivo remains limited. Consequently, biological effects observed with isolated compounds or extracts from other *Salvia* species should not be directly extrapolated to *S. nemorosa*. Future research should prioritize chemically characterized and standardized extracts or EOs, combined with appropriate positive controls, concentration-response analyses, and validated in vivo models where justified. Such studies should be complemented by a systematic toxicological assessment to establish safety profile and provide an appropriate basis for evaluating potential pharmacological applications.

Further phytochemical investigations could benefit from the integration of high-resolution LC-MS/MS, NMR-based metabolomics, preparative chromatography, and modern dereplication strategies. Combining these approaches with authenticated plant material and detailed reporting of extraction and analytical conditions would facilitate the discovery of previously uncharacterized metabolites while improving comparability among studies. In parallel, metabolomic and chemometric approaches applied to geographically and ecologically distinct populations could help resolve the contribution of genotype, habitat, environmental conditions, and developmental stage to the chemical phenotype of *S. nemorosa*.

Biotechnological approaches, including tissue culture and elicitation, may be useful for investigating and potentially enhancing the production of selected metabolites, as already demonstrated for rosmarinic acid in *S. nemorosa* cultures [33,34]. However, such approaches should be considered research strategies rather than evidence of therapeutic applicability. Future studies could extend the scope of these approaches to a wider range of metabolites and investigate the influence of elicitors, cultivation conditions, and metabolic regulation on their production. Such research could contribute to a better understanding of the biosynthetic capacity of *S. nemorosa* and provide controlled experimental systems for studying its chemical diversity.

Future research should move beyond isolated reports of individual compounds or biological activities toward integrated studies that connect plant identity, population and habitat characteristics, metabolite profiles, biological effects, and safety. This comprehensive approach would facilitate a more robust chemical and biological characterization of *S. nemorosa* and clarify which features are species-specific, environmentally variable, or shared with other *Salvia* species.

## 3. Materials and Methods

This narrative review was developed from literature identified in PubMed, Scopus, Web of Science, and Google Scholar, complemented by reference-list screening and targeted searches for comparative *Salvia* studies in which *S. nemorosa* data were reported separately. Priority was given to primary research providing species-specific information on phytochemical composition, compound isolation, essential-oil composition, cultivation or environmental effects on metabolite profiles, and biological activity. General reviews were used for contextual background and reference chasing rather than as primary evidence. Because the article is a critical narrative synthesis rather than a systematic or scoping review, PRISMA was not applied. The literature was interpreted comparatively, with particular attention to plant organ, geographical origin, population, cultivation conditions, extraction and analytical procedures, and the distinction between evidence obtained directly for *S. nemorosa* and effects reported for isolated constituents or other *Salvia* species.

## 4. Conclusions

*S. nemorosa* is a phytochemically diverse *Salvia* species with documented ethnomedicinal use and a body of experimental evidence supporting antioxidant, antimicrobial, enzyme-inhibitory, and selected cytotoxic activities in vitro. The synthesis of the available literature indicates that its chemical identity cannot be reduced to a single universal marker. Instead, recurrent phenolic constituents, including rosmarinic, sagerinic, and caffeoylquinic acids, coexist with sesquiterpene-rich but highly variable essential-oil profiles and with structurally diverse specialized metabolites such as quinones, diterpenoids, triterpenoids, and megastigmane glycosides. Comparative evidence further shows pronounced plasticity related to geographical origin, population, plant organ, developmental stage, cultivation and environmental conditions, and analytical methodology. Recent studies of drought, salinity, elicitation, and uniform interspecific cultivation reinforce this interpretation and additionally identify the high seed-oil and linolenic-acid content of *S. nemorosa* as a potentially distinctive feature deserving further investigation [35,47,48,49]. Biologically, *S. nemorosa* can show high antioxidant or enzyme-inhibitory activity under specific conditions, but current evidence does not establish general pharmacological superiority over other *Salvia* species. Its most defensible identity is therefore that of a chemically diverse and environmentally responsive taxon whose biological effects must be interpreted in relation to a defined population, plant organ, chemical profile, and experimental context. Future research should prioritize standardized sampling, comprehensive LC-MS/MS and NMR-based profiling, quantitative chemometric analysis of population-level variation, validated composition-activity relationships, toxicological evaluation, and appropriately designed in vivo studies.

## Figures and Tables

**Figure 1:**
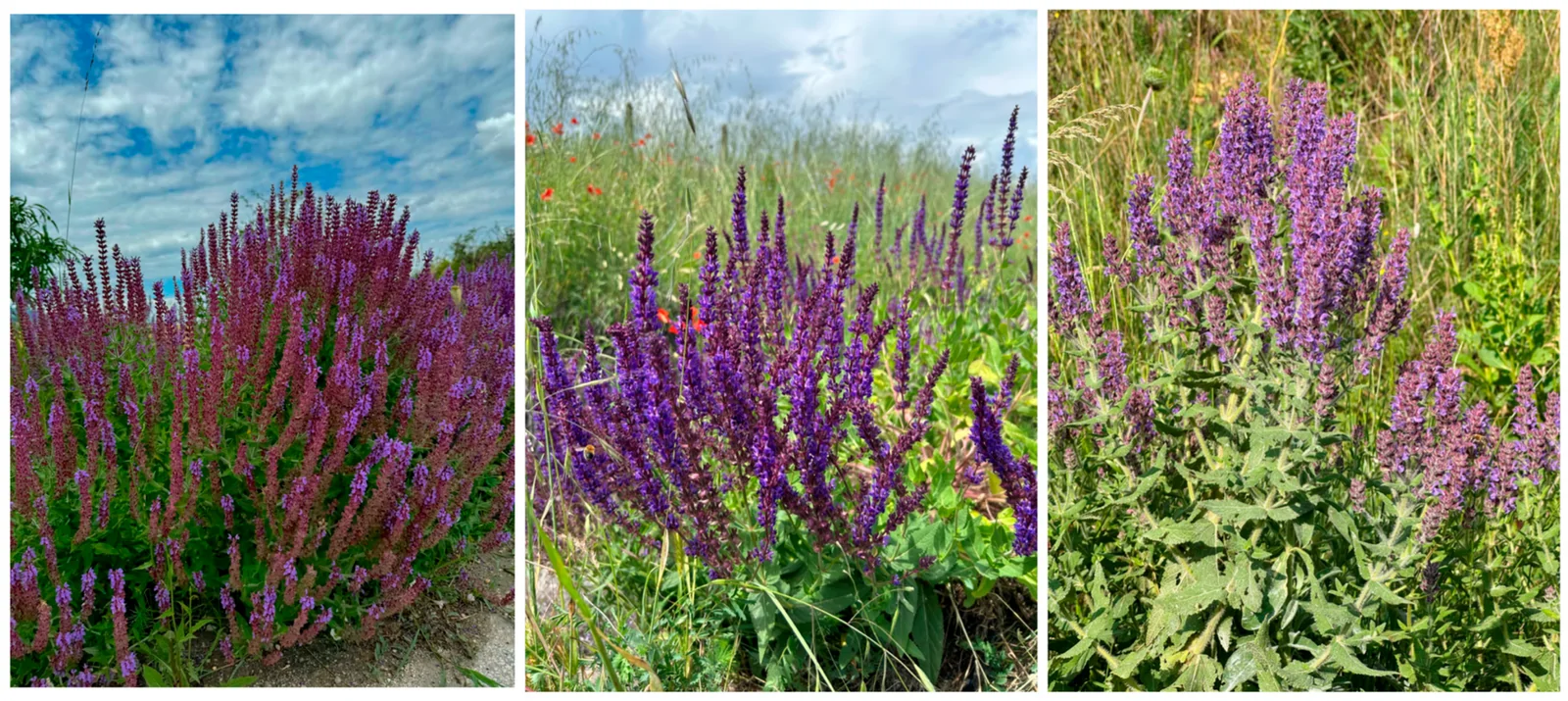
Wild populations of *S. nemorosa* L.

**Figure 2:**
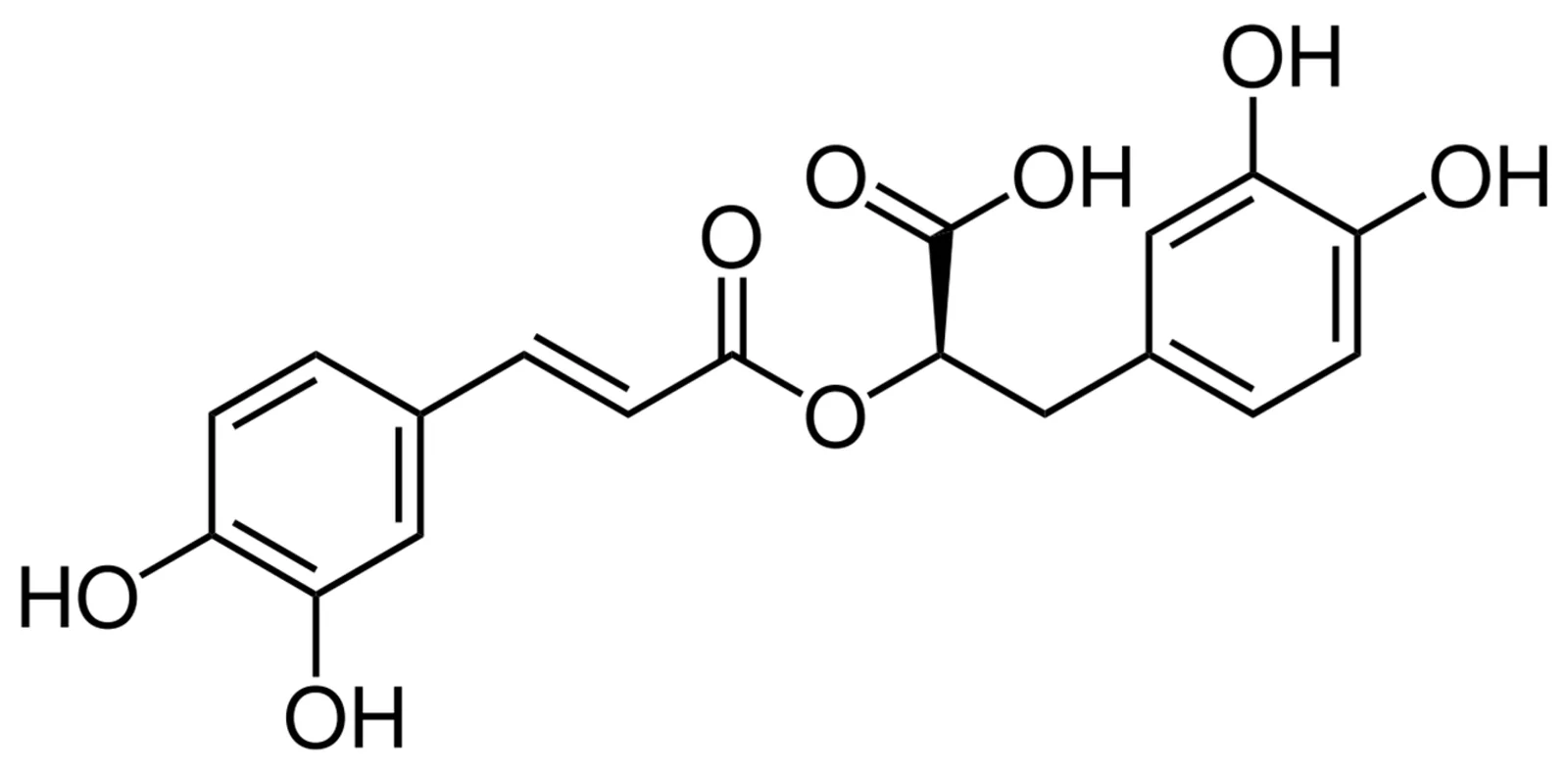
Chemical structure of rosmarinic acid ((2R)-3-(3,4-Dihydroxyphenyl)-2-{[(2E)-3-(3,4-dihydroxyphenyl)prop-2-enoyl]oxy}propanoic acid).

**Table 1:** Summary of studies on the phytochemical composition of *S. nemorosa* L. extracts from different geographical origins.

Methodological Approach	Main Findings	Country of Origin	Ref.
Chemical profiling of 70 *S. nemorosa* populations collected from 54 habitats. Methanolic extracts were analyzed using UHPLC-HRMS, the AlCl_3_ colorimetric assay, the Klein and Perry method for ascorbic acid determination, and UV–Vis spectrophotometry.	Total phenolic content (TPC) ranged from 33.87 to 66.24 mg GAE/g, total flavonoid content (TFC) from approximately 9.0 to 13.8 mg QE/g DW, and ascorbic acid content from 23 to 43 mg AA/g DW. Rosmarinic acid, sagerinic acid, and caffeoylquinic acids were the predominant phenolic compounds.	Iran	[13]
The chemical profile of *S. nemorosa* was evaluated using RP-HPLC, the Folin–Ciocalteu assay, and the AlCl_3_ colorimetric assay. Different extracts, including methanolic extract, were investigated.	Rosmarinic acid was the predominant compound (7584 µg/g extract). The methanolic extract showed the highest TPC (294 mg GAE/g extract) and TFC (117 mg QE/g extract).	Iran	[3]
Extracts of *S. nemorosa* were analyzed by UHPLC-LTQ Orbitrap MS for identification and quantification of the main phenolic compounds.	Extraction yields ranged from 3.03% to 15.75%. Epigallocatechin gallate and rosmarinic acid were identified as the predominant compounds.	Serbia	[12]
Methanolic extracts of *S. nemorosa* were analyzed by HPLC for identification and quantification of phenolic compounds and flavonoids.	Rosmarinic acid was the predominant phenolic acid (268.79 mg/100 g dried plant material), followed by caffeic acid (4.76 mg/100 g) and chlorogenic acid (4.75 mg/100 g). Apigenin (25.92 mg/100 g) and luteolin (9.86 mg/100 g) were the major flavonoids.	Romania	[14]
Bioassay-guided fractionation of a dichloromethane extract from the aerial parts of *S. nemorosa* was performed using VLC, CC, recrystallization, and NMR-based structural elucidation.	Clerodermic acid, a clerodane diterpenoid, was isolated and identified as an active antiproliferative constituent of *S. nemorosa*.	Iran	[18]
Methanolic extracts of leaves, stems, flowers, and roots of cultivated *S. nemorosa* were evaluated for total phenolic and flavonoid contents and rosmarinic acid.	Marked organ-dependent variation was observed. TPC ranged from 7.3 to 39.8 mg GAE/g extract and was highest in flowers, whereas TFC ranged from 68.8 to 141.9 mg QE/g extract and was highest in leaves. Rosmarinic acid content in leaves was 0.28 mg/g DW.	Iran	[42]
Methanolic extracts of *S. nemorosa* aerial parts were evaluated using UHPLC-MS, the Folin–Ciocalteu assay, and the AlCl_3_ spectrophotometric assay.	Rosmarinic acid was the predominant phenolic acid (1692.434 μg/g DW), followed by chlorogenic acid (56.316 μg/g DW). Lower levels of caffeic and ferulic acids were detected. Isoquercitrin was the predominant flavonoid (13.096 μg/g DW), followed by luteolin (2.178 μg/g DW) and rutin (1.014 μg/g DW).	Romania	[19]
Methanolic extracts of leaves and flowers of *Lamiaceae* species cultivated under Iranian climatic conditions were evaluated for total phenolic content.	Across the investigated species, TPC ranged from 6.87 to 33.22 mg GAE/g fresh weight. In *S. nemorosa*, leaves contained more polyphenols (11.96 mg GAE/g) than flowers (8.55 mg GAE/g).	Iran	[20]
Wild-growing *S. nemorosa* from the Mediterranean region was analyzed spectrophotometrically for total flavonoids, phenolic acids, tannins, and anthocyanins. Major phenolic constituents of the ethanolic leaf extract were identified and quantified by HPLC-DAD.	Phenolic acids were the predominant group in leaves (6.48%), whereas flavonoids and anthocyanins accounted for 0.88% and 0.06%, respectively. Rosmarinic acid was the major phenolic acid (14,200 µg/g), followed by chlorogenic (1900 µg/g), *p*-coumaric (1600 µg/g), and caffeic acid (300 µg/g). TPC ranged from 38.7 to 259.9 mg GAE/g plant extract.	Croatia	[21]
Fifty methanolic extracts prepared from 32 *Salvia* species collected from different regions of Iran were evaluated for total phenolic and flavonoid contents.	Among the investigated *S. nemorosa* samples, the population from West Azerbaijan showed the highest TPC and TFC, with values of 119.3 mg GAE/g plant extract and 51.8 mg CE/g plant extract, respectively.	Iran	[22]
The chemical profile of *S. nemorosa* was investigated by HPLC. Total phenolic and flavonoid contents were determined spectrophotometrically.	TPC and TFC were 40.2 mg GAE/g plant material and 1.18 mg RE/g plant material, respectively. Ferulic acid was the predominant phenolic acid (182.1 μg/g DW), followed by *p*-coumaric acid (146.4 μg/g DW). Rutin (263.8 μg/g DW), luteolin (67.5 μg/g DW), and apigenin (57.9 μg/g DW) were identified among the flavonoids.	Moldova	[23]

Abbreviations: AA, ascorbic acid; AlCl_3_, aluminium chloride; CC, column chromatography; CE, catechin equivalents; DW, dry weight; GAE, gallic acid equivalents; HPLC, high-performance liquid chromatography; HPLC-DAD, high-performance liquid chromatography with diode-array detection; NMR, nuclear magnetic resonance; QE, quercetin equivalents; RE, rutin equivalents; RP-HPLC, reversed-phase high-performance liquid chromatography; TFC, total flavonoid content; TPC, total phenolic content; UHPLC-HRMS, ultra-high-performance liquid chromatography–high-resolution mass spectrometry; UHPLC-MS, ultra-high-performance liquid chromatography–mass spectrometry; VLC, vacuum liquid chromatography.

**Table 2:** Comparative composition of the volatile constituents of *S. nemorosa* L. from different geographical origins.

Analytical Technique	Main Findings	Country of Origin	Ref.
GC-MS	**Aerial parts:** Pop. 1 (Perushtitsa): germacrene D (25.74%), β-caryophyllene (22.38%), sabinene (6.88%), caryophyllene oxide (5.77%). Pop. 2 (Asenovgrad): germacrene D (37.08%), β-caryophyllene (13.91%), 14-hydroxy-9-epi-(*E*)-caryophyllene (7.98%), caryophyllene oxide (7.35%). Pop. 3 (Ivan Vazovo): sabinene (21.89%), germacrene D (17.64%), β-caryophyllene (9.50%), bicyclogermacrene (7.67%), (*E*)-β-farnesene (6.90%). Pop. 4 (Slavovitsa): germacrene D (41.34%), β-caryophyllene (11.65%), phytol (5.21%).	Bulgaria	[43]
GC–MS	**Stems:** Pop. 1: hexadecanoic acid (59.8%), β-caryophyllene (12.1%), caryophyllene oxide (7.2%), germacrene D (7.1%), sabinene (5.2%). Pop. 2: β-caryophyllene (25.6%), caryophyllene oxide (25.2%), salvial-4(14)-en-1-one (13.9%), germacrene D (8.0%), sabinene (7.9%), β-bourbonene (6.1%), terpinen-4-ol (5.4%). Pop. 3: hexadecanoic acid (56.3%), β-caryophyllene (9.4%), germacrene D (6.6%). Pop. 4: hexadecanoic acid (58.1%), β-caryophyllene (8.9%). **Leaves:** Pop. 1: β-caryophyllene (34.0%), germacrene D (14.0%), caryophyllene oxide (13.2%), hexadecanoic acid (9.6%). Pop. 2: β-caryophyllene (30.4%), germacrene D (20.1%), caryophyllene oxide (19.5%). Pop. 3: hexadecanoic acid (24.4%), germacrene D (15.4%), β-caryophyllene (14.4%), sabinene (6.6%), caryophyllene oxide (5.3%). Pop. 4: β-caryophyllene (41.0%), germacrene D (28.3%), caryophyllene oxide (7.6%). Pop. 5: germacrene D (38.3%), β-caryophyllene (25.5%), caryophyllene oxide (5.4%). **Flowers:** Pop. 1: sabinene (43.0%), germacrene D (12.9%), β-caryophyllene (11.8%). Pop. 3: sabinene (43.1%), germacrene D (8.7%), γ-terpinene (8.4%), α-thujene (7.6%), β-caryophyllene (6.9%). Pop. 4: sabinene (36.6%), germacrene D (13.0%), β-caryophyllene (10.5%), α-thujene (5.9%), γ-terpinene (5.5%). Pop. 5: sabinene (44.1%), germacrene D (13.4%), β-caryophyllene (8.6%), α-thujene (5.3%).	Austria	[10]
GC-MS	**Aerial parts:** (*E*)-caryophyllene (26.37 ± 2.25%), phytol (16.92 ± 1.87%), germacrene D (15.34 ± 1.48%), sabinene (12.86 ± 1.12%), caryophyllene oxide (6.33 ± 1.63%).	Iran	[11]
GC-MS	**Flowers:** caryophyllene oxide (45.0%), spathulenol (23.0%), ledene oxide (9.2%), (*E*)-caryophyllene (7.0%), humulene epoxide (2.4%). **Leaves:** spathulenol (57.8%), caryophyllene oxide (28.2%), hexahydrofarnesyl acetone (3.8%), carvacrol (1.2%).	Iran	[3]
GC-FID and GC-MS	**Aerial parts:** caryophyllene oxide (23.4%), spathulenol (11.2%), γ-terpinene (8.6%), (*E*)-anethole (7.8%), humulene epoxide II (5.6%).	Serbia	[9]
GC-FID	**Aerial parts:** caryophyllene oxide (13.4%), β-caryophyllene (10.1%), germacrene D (5.7%).	Serbia	[8]
GC-MS	**Aerial parts:** β-caryophyllene (18.78–60.6%), caryophyllene oxide (19.0–27.0%), spathulenol (6.8–26.0%), bicyclogermacrene (7.65%), germacrene D (5.54–6.29%), linalool (5.00–8.11%), β-eudesmol (5.79%).	Iran	[44]
GC-MS	**Aerial parts:** sabinene (38.2%), β-caryophyllene (19.03%), germacrene D (18.62%).	Romania	[45]
GC–MS	**Aerial parts:** β-caryophyllene (41.6%), germacrene B (21.3%), caryophyllene oxide (6.8%), *cis*-β-farnesene (6.0%), germacrene D (5.6%), spathulenol (5.5%).	Iran	[46]
GC–MS	**Aerial parts:** Pop. 1 (Shazand): caryophyllene oxide (24.41%), spathulenol (18.17%), (*E*)-caryophyllene (12.07%), phytol (7.83%). Pop. 2 (Sangak): spathulenol (15.54%), caryophyllene oxide (10.59%), 14-hydroxy-9-epi-(*E*)-caryophyllene (8.58%), (*E*)-caryophyllene (6.82%). Pop. 3 (Polor): thymol (17.59%), caryophyllene oxide (10.44%), *p*-cymene (5.08%), spathulenol (3.87%). Pop. 4 (Tehran): (*E*)-caryophyllene (27.38%), α-terpineol (14.25%), germacrene D (10.45%), β-pinene (6.87%). Pop. 5 (Kerman): spathulenol (25.7%), phytol (20.4%), 14-hydroxy-9-epi-(*E*)-caryophyllene (8.39%), (*E*)-caryophyllene (7.33%). Pop. 6 (Amir Kabir): spathulenol (37.13%), caryophyllene oxide (16.36%), 14-hydroxy-9-epi-(*E*)-caryophyllene (4.28%).	Iran	[17]
GC-FID and GC-MS	**Aerial parts:** aromadendrene (47.5%), tetramethylbutane (18.12%), caryophyllene oxide (13.48%).	Iran	[42]

Abbreviations: GC–FID, gas chromatography–flame ionization detection; GC–MS, gas chromatography–mass spectrometry; Pop., population.

**Table 3:** In vitro biological activities reported for *S. nemorosa* extracts. Values are reported as given in the original studies; assay conditions should be considered when comparing results.

Plant Material	Country of Origin	Study Design	Main Findings	Ref.
Flowering branches	Iran	Aqueous, methanolic, and hydroalcoholic extracts at different concentrations were evaluated for antimicrobial activity using agar diffusion assays.	The methanolic extract showed the highest activity against *Staphylococcus aureus* (inhibition zone: 14 ± 1 mm), whereas the aqueous extract was the most active against *Pseudomonas aeruginosa* (26 ± 1 mm). No activity against *Proteus vulgaris* was observed.	[24]
Aerial parts	Iran	Extracts obtained with *n*-hexane, dichloromethane, and methanol were evaluated for antioxidant activity (DPPH and FRAP), antimicrobial activity (disk diffusion and MIC assays), enzyme-inhibitory activity, and cytotoxicity.	The methanolic extract exhibited the strongest DPPH radical-scavenging activity (IC_50_ = 82 µg/mL). Moderate-to-high inhibitory activity against xanthine oxidase, aldehyde oxidase, α-glucosidase, and acetylcholinesterase was observed, together with moderate cytotoxicity. Gram-negative bacteria were only moderately inhibited by the methanolic extract compared with Gram-positive bacteria.	[3]
Aerial parts	Iran	The antioxidant activity of a methanolic extract was evaluated using the DPPH radical-scavenging assay.	The extract showed DPPH radical-scavenging activity with an IC_50_ value of 40.22 µg/mL, indicating lower activity than the reference standards.	[11]
Inflorescences	Serbia	Fourteen extracts obtained using different solvent systems, including ethanol-water, ethanol, dichloromethane-hexane, and butanol, were evaluated for biological activity.	Most extracts showed no cytotoxic effects on human keratinocytes. The ethanol-butanol (50:50) extract showed the strongest wound-healing activity, whereas ethanol-water extracts exhibited the highest antioxidant potential. Butanol extraction enhanced enzyme-inhibitory and antimicrobial activities. The most active extracts showed >90% inhibition of *Staphylococcus lugdunensis* biofilm formation.	[12]
Aerial parts	Moldova	Antioxidant activity was assessed using DPPH, ABTS, and FRAP assays. Antimicrobial activity was evaluated against standard microbial strains, and cytotoxicity was investigated using MCF-7, HL60, and K562 cell lines.	*S. nemorosa* showed relatively low antioxidant activity compared with the other investigated species. MIC values were 2.5 mg/mL against *S. aureus* ATCC 25923 and *S. pneumoniae* ATCC 49619, 5 mg/mL against *E. coli*, 2.5 mg/mL against *P. aeruginosa* ATCC 27853, and 21.25 mg/mL against *C. albicans* ATCC 10231. IC_50_ values against MCF-7, HL60, and K562 cells were generally >50 µg/mL, and the extract was reported as non-cytotoxic in these cell lines.	[25]
Leaves	Hungary	The lipid peroxidation-inhibitory activity of a 50% aqueous methanolic extract was evaluated.	*S. nemorosa* showed antioxidant activity, with an EC_50_ value of 123.8 µg/mL, and was reported to be more active than the reference compound ascorbic acid.	[26]
Leaves	Hungary	Antioxidant activity was investigated using enzyme-dependent and enzyme-independent lipid peroxidation systems.	*S. nemorosa* extracts demonstrated considerable concentration-dependent antioxidant activity.	[27]
Aerial parts (dichloromethane extract; bioassay-guided isolated clerodermic acid)	Iran	The antiproliferative activity of isolated clerodermic acid against human A549 lung cancer cells was assessed using the MTT assay, Annexin V/PI apoptosis detection, DNA fragmentation analysis, DAPI staining, cell-cycle analysis, and qPCR.	Clerodermic acid exhibited antiproliferative activity against A549 cells (IC_50_ = 35 μg/mL), accompanied by cytotoxic/genotoxic effects, induction of apoptosis, and inhibition of HIF-1α expression under hypoxic conditions.	[18]
Leaves, stems, flowers, and roots	Iran	Methanolic extracts from different plant organs were evaluated for antioxidant activity using the DPPH radical-scavenging assay.	IC_50_ values ranged from 14.3 to 90.7 μg/mL. The stem extract showed the strongest radical-scavenging activity.	[42]
Aerial parts	Romania	The antioxidant activity of a methanolic extract was evaluated using DPPH and ABTS assays.	Among the investigated *Salvia* species, *S. nemorosa* showed the strongest DPPH radical-scavenging activity (IC_50_ = 15.84 μg/mL). The ABTS assay confirmed its antioxidant potential (IC_50_ = 22.71 μg/mL).	[19]
Aerial parts	Iran	Cytotoxicity against HL60, K562, and MCF-7 cell lines, antioxidant activity using the DPPH assay, and antimicrobial activity using agar disk diffusion and broth microdilution methods were evaluated.	The 80% methanolic extract showed moderate cytotoxicity against K562 cells (IC_50_ = 87.0 ± 6.7 µg/mL) but was inactive against HL60 and MCF-7 cells. DPPH radical-scavenging activity was observed (IC_50_ = 138.43 µg/mL). Antimicrobial activity was weak, with activity reported only against *S. typhi* (MIC = 10 mg/mL).	[28]
Aerial parts	Iran	The antioxidant properties of a methanolic extract of *S. nemorosa* were evaluated by comparison with 14 other Lamiaceae species cultivated under Iranian climatic conditions.	Among the investigated *Salvia* species (*S. nemorosa*, *S. officinalis*, and *S. sclarea*), *S. nemorosa* showed the highest antioxidant potential in the FRAP assay. Leaf extracts showed higher antioxidant capacity (2.92 mmol Fe/100 g fresh weight) than flower extracts (2.66 mmol Fe/100 g fresh weight).	[20]
Leaves	Croatia	The antioxidant properties and mechanisms of action of an ethanolic extract were evaluated using five analytical assays. α-Glucosidase inhibitory activity was also investigated.	The extract showed significant concentration-dependent DPPH and NO radical-scavenging activities and reducing capacity. At 200 µg/mL, it inhibited lipid peroxidation by 39%. The extract also inhibited α-glucosidase by 39%, compared with 89% inhibition by acarbose.	[21]
Leaves and roots	Iran	The antidiabetic potential of methanolic extracts was evaluated by determining their inhibitory effects on α-amylase and α-glucosidase and comparing them with extracts from 13 other Lamiaceae species.	The root extract showed strong α-amylase inhibitory activity (0.270 mmol ACAE/g extract), whereas the leaf extract showed lower activity (0.170 mmol ACAE/g extract). In the α-glucosidase assay, root and leaf extracts showed moderate inhibitory activities of 2.72 and 2.96 mmol ACAE/g extract, respectively.	[29]
Aerial parts	Iran	Hydromethanolic extracts of 50 *Salvia* samples representing 32 species were evaluated for α-glucosidase inhibitory activity.	The 80% methanolic extracts of *S. nemorosa* collected from four Iranian regions showed α-glucosidase inhibition ranging from 23.5% to 70.1%. The West Azerbaijan sample showed the strongest inhibition (70.1% at 1000 µg/mL), compared with 87.8% for the reference inhibitor acarbose.	[30]

Abbreviations: ABTS, 2,2′-azino-bis(3-ethylbenzothiazoline-6-sulfonic acid); ACAE, acarbose equivalent; DAPI, 4′,6-diamidino-2-phenylindole; DPPH, 2,2-diphenyl-1-picrylhydrazyl; EC_50_, half-maximal effective concentration; FRAP, ferric reducing antioxidant power; HIF-1α, hypoxia-inducible factor 1-alpha; IC_50_, half-maximal inhibitory concentration; MIC, minimum inhibitory concentration; MTT, 3-(4,5-dimethylthiazol-2-yl)-2,5-diphenyltetrazolium bromide; NO, nitric oxide; PI, propidium iodide; qPCR, quantitative polymerase chain reaction.

**Table 4:** Summary of the biological activities of *S. nemorosa* essential oils.

Plant Material	Country of Origin	Study Design	Results	Ref.
Leaves	Serbia	The antioxidant activity of *S. nemorosa* essential oil (EO) was evaluated using the DPPH radical-scavenging assay, hydroxyl radical-scavenging assay (deoxyribose method), and lipid peroxidation inhibition assays. Antimicrobial activity was assessed using the microdilution technique and hole-plate agar diffusion method.	The EO exhibited antioxidant activity in the DPPH radical-scavenging assay (IC_50_ = 0.98 μL/mL) and hydroxyl radical-scavenging assay (IC_50_ = 0.11 μL/mL). Lipid peroxidation was inhibited in both the Fe^2+^/ascorbate (IC_50_ = 0.58 μL/mL) and Fe^2+^/H_2_O_2_ (IC_50_ = 0.01 μL/mL) systems. Notable antifungal activity was observed, with MIC values ranging from 100.0 to 200.2 µL/mL and MFC values from 200.0 to 400.2 µL/mL. The EO showed stronger activity against Gram-positive bacteria, whereas Gram-negative bacteria were less susceptible. Activity was observed only against *S. Typhi* and *Shigella sonnei* among the tested Gram-negative bacteria, while no activity was detected against *Pseudomonas aeruginosa*, *Escherichia coli*, or *Salmonella Enteritidis*.	[64]
Aerial parts	Iran	The antimicrobial activity of *S. nemorosa* EO was evaluated against *Staphylococcus aureus*, *Staphylococcus epidermidis*, *Escherichia coli*, and *Candida albicans* using disk diffusion and serial dilution methods.	The EO exhibited moderate antimicrobial activity. The MIC and MBC/MFC values were 6.25 and 12.5 mg/mL against *S. aureus*, 6.25 and 12.5 mg/mL against *S. epidermidis*, 12.5 and 25 mg/mL against *E. coli*, and 3.125 and 6.25 mg/mL against *C. albicans*, respectively.	[11]
Aerial parts	Iran	The antioxidant activity of *S. nemorosa* EOs obtained by conventional hydrodistillation (HD) and ultrasound-assisted hydrodistillation (UAHD) was evaluated using the DPPH radical-scavenging assay.	The EO obtained by UAHD showed stronger DPPH radical-scavenging activity (34.15% inhibition) than that obtained by conventional HD (13.49% inhibition).	[31]

Abbreviations: DPPH, 2,2-diphenyl-1-picrylhydrazyl; EO, essential oil; HD, hydrodistillation; IC_50_, half-maximal inhibitory concentration; MBC, minimum bactericidal concentration; MFC, minimum fungicidal concentration; MIC, minimum inhibitory concentration; UAHD, ultrasound-assisted hydrodistillation.

## Data Availability

Data are contained within the article.

## Abbreviations

The following abbreviations are used in this manuscript:

CQAs	Caffeoylquinic acids
EO	Essential oil
AA	Ascorbic acid
AlCl_3_	Aluminium chloride
HD	Hydrodistillation
UAHD	Ultrasound-assisted hydrodistillation
DPPH	2,2-diphenyl-1-picrylhydrazyl
ABTS	2,2′-azino-bis(3-ethylbenzothiazoline-6-sulfonic acid)
FRAP	Ferric Ion Reducing Antioxidant Power Assay
MTT	3-(4,5-dimethylthiazol-2-yl)-2,5-diphenyltetrazolium bromide
HPLC	High-Performance Liquid Chromatography
HPLC-DAD	High-Performance Liquid Chromatography- Diode-Array Detector
UHPLC-MS	Ultra-High-Performance Liquid Chromatography–Mass Spectrometry
RP-HPLC	Reversed-phase High-Performance Liquid Chromatography
GC-MS	Gas Chromatography–Mass Spectrometry
GC-FID	Gas Chromatography–Flame Ionization Detector
VLC	Vacuum liquid chromatography
CC	Column chromatography
TFC	Total flavonoid content
TPC	Total phenolic content
DW	Dry weight
GAE	Gallic acid equivalents
QE/g DW	Quercetin Equivalent per gram of dry weight
GAE/g	Gallic Acid Equivalent per gram
EC_50_	Half-maximal effective concentration
IC_50_	Half-maximal inhibitory concentration
HIF-1α	Hypoxia-inducible factor 1-alpha
MIC	Minimum Inhibitory Concentration
MBC	Minimum Bactericidal Concentration
MFC	Minimum Fungicidal Concentration
NO	Nitric oxide
PI	Propidium iodide
qPCR	Quantitative polymerase chain reaction
DAPI	4′,6-diamidino-2-phenylindole
CE	Catechin equivalent
ACAEs	Acarbose equivalents
